# The potential of broadly neutralizing antibodies for HIV prevention

**DOI:** 10.1002/jia2.26257

**Published:** 2024-05-17

**Authors:** Huub C. Gelderblom, Lawrence Corey, Dan H. Barouch

**Affiliations:** ^1^ Vaccine and Infectious Disease Division Fred Hutchinson Cancer Center Seattle Washington USA; ^2^ Department of Medicine University of Washington Seattle Washington USA; ^3^ Beth Israel Deaconess Medical Center Boston Massachusetts USA

1

The number of new HIV acquisitions globally has declined, but not rapidly enough to meet the 2030 targets set by UNAIDS and the United Nations Sustainable Development Goals (SDGs) [1, 2]. Despite intense efforts such as those to support the UNAIDS 95‐95‐95 targets and to expand the availability of oral pre‐exposure prophylaxis (PrEP), progress in primary prevention of HIV acquisition has lagged. There were 1.3 million new HIV acquisitions in 2022, and at current rates of decline, this number is projected to decrease to 900,000 new HIV acquisitions by 2030, which is far from the SDG target of 300,000. The number of people living with HIV will continue to increase from 39 million in 2022 to a projected 45 million in 2030 [1, 2, 3].

In this Viewpoint, we review the potential of HIV broadly neutralizing monoclonal antibodies (bnAbs) as a long‐acting injectable immunoprophylaxis regimen to reduce HIV acquisition in high‐risk populations. HIV bnAbs can recognize and neutralize a wide range of HIV strains, making them a promising tool for HIV prevention [4]. In the last 10−15 years, several HIV bnAbs have been isolated and have entered clinical development [5] (Table 1). These include antibodies against the CD4 binding site, the V3 glycan supersite and the V2 apex of the Env trimer. During the COVID‐19 pandemic, monoclonal antibodies were delivered on an unprecedented scale for the prevention of SARS‐CoV‐2, showing the feasibility of using antibodies for prevention.

**Table 1:** Completed and ongoing clinical trials for HIV bnAbs‐mediated prevention in adults

		CD4 binding site							MPER	V2			V3				
Trial registration number VRC01	Protocol VRC01LS	VRC07‐523LS	VRC01.23LS	N6LS	3BNC117	3BNC117LS	10E8VLS	PGDM1400	PGDM1400LS	CAP256V2LS	PGT121	PGT121.414.LS	10‐1074	10‐1074LS			Countries
NCT02165267	HVTN 104	X															USA
NCT02568215	HVTN 703/HPTN 081 (AMP)	X															Botswana, Kenya, Malawi, Mozambique, South Africa, Tanzania and Zimbabwe
NCT02716675	HVTN 704/HPTN 085 (AMP)	X															Brazil, Peru, Switzerland and USA
NCT02797171	HVTN 116	X	X														South Africa and USA
NCT03387150	HVTN 127/HPTN 087			X													Switzerland and USA
NCT03735849	HVTN 128			X													USA
NCT03928821	HVTN 130/HPTN 089			X									X				USA
NCT04212091	HVTN 136/HPTN 092			X										X			USA
NCT05184452	HVTN 140/HPTN 101			X							X			X			Kenya, South Africa, USA and Zimbabwe
NCT01993706	VRC 602	X															USA
NCT03015181	VRC 605			X													USA
NCT02599896	VRC 606		X														USA
NCT03538626	VRC 609					X											USA
NCT03565315	VRC 610			X					X								USA
NCT04408963	VRC 611											X					USA
NCT05627258	VRC 615				X												USA
NCT02960581	IAVI T001												X				USA
NCT03205917	IAVI T002									X			X				USA
NCT03721510	IAVI T003			X						X			X				USA
NCT02018510	RU MCA‐0835						X										Germany and USA
NCT02511990	RU MCA‐0885														X		Germany and USA
NCT02824536	RU YCO‐0899						X								X		USA
NCT03254277	RU YCO‐0946							X									USA
NCT03554408	RU YCO‐0971							X								X	USA
PACTR202112683307570	CAPRISA 012A			X									X				South Africa
PACTR202112683307570	CAPRISA 012B			X									X				South Africa
PACTR202112683307570	CAPRISA 012C			X													South Africa

The proof‐of‐concept that an HIV bnAb can prevent HIV acquisition was demonstrated in 2021 by the Antibody Mediated Prevention (AMP) trials [6]. These two harmonized phase 2B clinical trials—one conducted in the United States and Latin America in men who have sex with men and transgender persons and the other conducted in sub‐Saharan Africa in cisgender women—showed that the prototype HIV bnAb VRC01 could prevent HIV acquisition, but was only effective against sensitive virus (IC80 < 1 µg/ml). The determinant of efficacy was the susceptibility of the infecting HIV strain to the antibody. The trials also provided a target serum antibody titre as a correlate of protection [7]. For HIV bnAbs to achieve broad protection against circulating HIV strains, a combination of antibodies targeting multiple epitopes will be needed. Several groups have shown that a cocktail of three complementary bnAbs, such as a combination of antibodies targeting the CD4 binding site, V3 loop and V2 loop, provide broad neutralization coverage of global viruses in vitro, which supports the rationale for clinical evaluation of such bnAb cocktails [8, 9].

Next‐generation HIV bnAbs have entered clinical trials [5, 10] (Table 1). These antibodies have been engineered to include mutations in the variable Fab region for greater potency and breadth, as well as mutations M428L/N434S or “LS” in the constant Fc region to extend antibody half‐life in vivo, allowing administration every 6 months [11].

HIV bnAbs would complement existing PrEP strategies and would increase choices for HIV prevention. Despite significant strides in antiretroviral treatment for prevention (undetectable equals untransmittable; U = U), progress in HIV prevention has been limited. Oral PrEP was approved over a decade ago but remains underutilized with only 6.2 million current users [12], compared with 18.4 million new HIV acquisitions since 2012 [3]. Many people discontinue PrEP shortly after initiation [13], although targeted campaigns in high‐income areas like Amsterdam have shown success [14]. In sub‐Saharan Africa and other regions, lack of access and stigma may hinder PrEP uptake, particularly among young women [13].

Diverse and accessible HIV prevention methods are needed to accommodate individual preferences and to increase prevention coverage. The concept of Number Needed to Treat suggests tens to hundreds of millions of people would need to use prevention strategies to make a substantial dent in HIV incidence [15]. HIV incidence rates fluctuate between regions, ranging from increases in Brazil and Peru to a slowing decline in South Africa, highlighting the complexity of this challenge [3]. Innovations in long‐acting injectable PrEP, such as cabotegravir [16, 17] and lenacapavir, alongside the development of HIV bnAbs, present promising alternatives to daily pills for HIV prevention. Cabotegravir requires injections every 2 months, and lenacapavir and HIV bnAbs are anticipated to require administration every 6 months, which may provide particularly convenient options.

In conclusion, the development of long‐acting prevention methods, including PrEP and bnAbs, is critical for control of the global HIV pandemic. As clinical studies of HIV bnAbs move forward, we must also address the social, economic and structural barriers to delivering HIV prevention and treatment at scale. Success will require a concerted and collaborative effort across all sectors of society, including governments, pharmaceutical companies, healthcare providers, communities and individuals to make HIV bnAbs globally available. As we look beyond 2030, the integration of innovative scientific tools with equitable access policies will be critical for turning the tide against HIV/AIDS.

## COMPETING INTERESTS

The authors declare no competing interests.

## AUTHORS’ CONTRIBUTIONS

HCG, LC and DHB contributed to writing this manuscript.
